# Expression and Function Analysis of Mitotic Checkpoint Genes Identifies TTK as a Potential Therapeutic Target for Human Hepatocellular Carcinoma

**DOI:** 10.1371/journal.pone.0097739

**Published:** 2014-06-06

**Authors:** Xiao-Dong Liang, Yue-Chu Dai, Zhao-Yun Li, Mei-Fu Gan, Shi-Rong Zhang, Yin-Pan, Hong-Sheng Lu, Xue-Quan Cao, Bei-jia Zheng, Ling-Fen Bao, Dan-Dan Wang, Li-Ming Zhang, Sheng-Lin Ma

**Affiliations:** 1 Affiliated Hangzhou Hospital of Nanjing Medical University, Hangzhou, First People’s Hospital, Hangzhou, China; 2 Central Hospital of Taizhou City, Taizhou, Zhejiang, China; 3 Taizhou Hospital, Wenzhou Medical University Linhai, Zhejiang, China; Virginia Commonwealth University, United States of America

## Abstract

The mitotic spindle checkpoint (SAC) genes have been considered targets of anticancer therapies. Here, we sought to identify the attractive mitotic spindle checkpoint genes appropriate for human hepatocellular carcinoma (HCC) therapies. Through expression profile analysis of 137 selected mitotic spindle checkpoint genes in the publicly available microarray datasets, we showed that 13 genes were dramatically up-regulated in HCC tissues compared to normal livers and adjacent non-tumor tissues. A role of the 13 genes in proliferation was evaluated by knocking them down via small interfering RNA (siRNA) in HCC cells. As a result, several mitotic spindle checkpoint genes were required for maintaining the proliferation of HCC cells, demonstrated by cell viability assay and soft agar colony formation assay. Then we established sorafenib-resistant sublines of HCC cell lines Huh7 and HepG2. Intriguingly, increased TTK expression was significantly associated with acquired sorafenib-resistance in Huh7, HepG2 cells. More importantly, TTK was observably up-regulated in 46 (86.8%) of 53 HCC specimens. A series of *in vitro* and *in vivo* functional experiment assays showed that TTK overexpression promoted cell proliferation, anchor-dependent colony formation and resistance to sorafenib of HCC cells; TTK knockdown restrained cell growth, soft agar colony formation and resistance to sorafenib of HCC cells. Collectively, TTK plays an important role in proliferation and sorafenib resistance and could act as a potential therapeutic target for human hepatocellular carcinoma.

## Introduction

Human hepatocellular carcinoma (HCC) has been considered a tumor highly insensitive to conventional chemotherapy [1]. In the past, there no well-established effective adjuvant therapy but surgical or topical therapy [2]. However, targeted molecular therapies provide significant benefits in patients with HCC. Sorafenib (Nexavar), an oral multikinase inhibitor with activity against Raf-1, B-Raf, VEGFR2, PDGFR and c-Kit receptors, has shown anti-tumor effects on HCC patients [3]–[5]. And sorafenib is the only clinically approved drug and considered the standard HCC treatment [6], [7]. However, many patients may develop acquired resistance to sorafenib, so its clinical benefits remain modest. Therefore, it is urgent to identify therapeutic biomarkers to improve the treatment response in HCC.

The spindle assembly checkpoint (SAC), also referred to as the mitotic checkpoint or M-phase checkpoint, controls cell cycle progression and is normally responsible for correct alignment of all chromosomes and proper attachment to the mitotic spindle [8], [9]. Recently, more and more genes which play a role in spindle assembly checkpoint have been identified through a variety of experiment and computed approaches. These spindle assembly checkpoint genes were shown to be associated with chromosomal instability (CIN) and aneuploidy, the common abnormalities in human cancers. More importantly, altered expression or mutations of mitotic checkpoint genes have been detected in some cancers. For example, the expression of MAD2 gene decreases in breast carcinoma [10] and mutant alleles of BUB1 gene mutation occurs in colorectal carcinoma [11]. In addition, inhibition of the mitotic checkpoint is lethal to human cancer cells, and has therapeutic potential in cancer treatment [12], [13].

The impairment of spindle assembly checkpoint frequently occurred in HCC with CIN [14]. However, recent researches on the whole genomes or exomes sequencing of HCC specimens show that somatic mutations in mitotic checkpoint genes were infrequent in hepatocellular carcinoma [15], [16]. In this study, we supposed that mitotic spindle checkpoint genes are largely altered at the transcriptional level in human hepatocellular carcinoma. We comprehensively examined the expression profile of 137 selected genes known to be involved in various molecular mechanisms associated with mitotic spindle checkpoint, by means of large-scale analysis of gene expression from public HCC microarray datasets. Among 13 marked up-regulated genes in HCC patients, we demonstrated that TTK gene, encoding a dual specificity protein kinase essential for chromosome alignment at the centromere during mitosis and required for centrosome duplication, is a potential therapeutic target for HCC cells resistant to sorafenib.

## Materials and Methods

### Reagents and Antibodies

Sorafenib was purchased from Selleck chemicals and 5-Flurouracil (5-Fu), 2,4-dihydroxy-5-fluoropyrimidine was obtained from the Sigma-Aldrich Chemical Co (St.Louis, MO, USA). For in vitro experiments, both drugs were dissolved in pure DMSO. Controls were treated with DMSO concentrations of the highest combination groups (maximum 0.3% DMSO). Antibodies for immunoblotting were purchased from Santa Cruz Biotechnology (Santa Cruz, CA, USA).

### Tissue Specimens and Cell Lines

This study was approved by the ethics committee of the affiliated Hangzhou Hospital of Nanjing Medical University. Written informed consent was obtained from each subject prior to the use of their tissue for scientific research. Tumor and non-tumorous liver tissues from surgical specimens were frozen in liquid nitrogen immediately after surgical resection and stored in liquid nitrogen until use. Tumor samples were confirmed to be hepatocellular carcinoma. Huh7 (JCRB0403, Japan) and HepG2 (HB-8065, ATCC, VA) cell lines were cultured in Dulbecco’s modified Eagle’s medium supplemented with 10% fetal bovine serum (FBS), 100 units/mL penicillin, and 100 mg/mL streptomycin in a 5% CO_2_-humidified chamber at 37°C.

### Establishment of Sorafenib-resistant HCC Cell Sublines

The sorafenib-resistant Huh7 and HepG2 cell lines were established as previously described [17], [18]. Briefly, when Huh7 and HepG2 cells were growing exponentially, they were exposed to sorafenib at escalating concentrations (0.5, 1, 2, 5, 10 and 15 µM). In the presence of different concentrations of sorafenib, the dead cells were washed out with phosphate-buffered saline (PBS) and the minority of treated cells gradually acquired the sorafenib-resistance. Then the resistant subclones were isolated by limiting dilution. The sorafenib-resistant subclones was established 5 months after the treatment was initiated. These cell sublines (Huh7R and HepG2R) were grown in the medium with 0.1 µM sorafenib for maintenance of the acquired sorafenib-resistant phenotype, and before Huh7R and HepG2R cell sublines were used to experimental test, they were subcultured at least 3 times in a sorafenib-free medium.

### In silico Gene Expression Analysis

The 137 selected mitotic checkpoint genes were in **Table S1**, and microarray expression datasets from Gene Expression Omnibus (http://www.ncbi.nlm.nih.gov/geo/) were listed in **Table S2** and analyzed using the Expression Profiler Software (http://www.ebi.ac.uk/expressionprofiler). The log2-transformed expression values from various datasets were subject to median-normalization when multiple probes were converted to the same Entrez GeneID, signal intensities were averaged to obtain single values for each Entrez GeneID. The significance for microarrays analysis was performed using Student’s *t* test.

### Plasmids Construction

The open reading frame (ORF) of TTK (NM_003318) was amplified by high-fidelity PCR from the cDNA pool of HCC patients and inserted into the FLAG-tagged pcDNA3.1 vector. For the construct of RNAi plasmid, a specific oligonucleotide fragment transcribing short hairpin RNA (shRNA) for TTK silence was subcloned into the pSUPER vector with neomycin resistant gene (OligoEngine). The oligonucleotides for TTK silennce were synthesized as follows: forward, 5′-*GATCCCC* CCGGAACGAAATAGCTTAT *TTCAAGAGA* ATAAGCTATT TCGTTCCGG *TTTTTGGAAA*-3′, reverse, 5′-*AGCTTTTCCAAAAA* CCGGAACGAAATAGCTTAT *TCTCTTGAA* ATAAGCTATT TCGTTCCGG *GGG*-3′ The pSUPER vector containing irrelevant nucleotide was used as negative control.

### Cell Viability and Cytotoxicity Assay

HCC cells were seeded in 96-well flat-bottomed plates (4,000 per well) in DMEM with 10% FBS and incubated overnight at 37°C in the culture incubator. On the following day, the medium was replaced with fresh medium containing sorafenib or 5-FU. Treatment with sorafenib was done for 48 h at 1 µM and that with 5-FU was for 48 h at 4 mg/L. Cell viability or cytotoxicity was evaluated using Cell Counting Kit-8 (CCK-8) according to the manufacturer’s instructions. Briefly, add 10 µl of CCK-8 solution to each well, and then incubate for 2 h at 37°C. The cell viability was reflected by the absorbance value at 450 nm filter. All experiments were independently repeated 3 times.

### RNA Interference (RNAi)

Small interference RNAs (siRNAs) targeting the 13 markedly up-regulated mitotic spindle checkpoint genes were chemically synthesized (GenePharma), and were dissolve overnight to 20 µM solution. 3 different siRNAs for each gene were pooled and these siRNAs were delivered into HCC cells with Lipofectamine (Invitrogen). The targeting sequences of these siRNAs were listed in **Table S3**.

### Reverse Transcription (RT) and PCR

Total RNA was extracted by using TRIzol Reagent (Invitrogen Life Technologies) according to the manufacturer’s instructions. Extracted RNA was quantitated by NanoDrop 2000 (Thermo Fisher Scientific, Waltham, MA). Reverse transcription reaction was performed using M-MLV reverse transcriptase (Promega). For the quantitative real-time PCR, the relative mRNA level of TTK was normalized to β-actin in each sample. All reactions were performed in triplicate using Thermal Cycler Dice Real Time System and SYBR green dye (TaKaRa). For the semi-quantitative PCR, the amplified products were observed by electrophoresis on 2% agarose gel and visualized after staining with ethidium bromide, where β-actin was used as loading control. The primers of TTK were shown in **Table S3**.

### Western Blot Assay

Total protein samples were prepared in lysis buffer [25 mmol/L Tris (pH 6.8), 1% SDS, 5 mmol/L EDTA, protease inhibitor cocktail (Sigma)] and subjected to gel electrophoresis using 10% SDS-PAGE and transferred onto nitrocellose membrane. The blot is incubated with blocking solution (5% nonfat milk and 0.1% Tween 20 in PBS) for 2 h at room temperature, and then with anti-TTK antibody by 1∶100 dilutions at room temperature overnight, and then with secondary antibody for 40 minutes. The immunostaining signal of TTK protein was visualized with Odyssey infrared imaging system (LI-COR).

### Clonogenicity Assay

HCC cells were cultured in the medium containing 0.6–1 mg/mL G418. After 3∼4 weeks, the remaining colonies were washed twice with PBS, stained with crystal violet. For the soft ager colony formation assay, transfected cells were grown in medium containing 1% base agar and 0.5% top agar. The forming colonies were stained by crystal violet and then counted according to defined size of colony. All experiments were independently repeatedly at least 3 times. Statistical significance of colony number difference was calculated by two-tailed Student’s *t* test.

### Establishment of Stable Cell Lines

To establish stably TTK-expressing HCC cells, Huh7 cells were transfected with the recombinant plasmid pcDNA3.1-TTK, then transfected cells were exposed to selection in medium containing 0.6 mg/ml G418 (Life Technologies Inc.). These cells were cultured under selective medium every 3 days until G418-resistant colonies grow. In addition, the established sorafenib-resistant Huh7R cells were transfected with pSUPER-shTTK plasmid and subject to G418-selection. And then some colonies were selected for further identification of TTK expression.

### Apoptosis Assay

Annexin V/PI apoptosis assay kit was used according to the protocol provided by the manufacturer (BD Biosciences Pharmingen). The HCC cells were harvested and washed twice with cold PBS, then resuspended in binding buffer at a concentration of 1×10^6^ cells/ml. Fluorescein isothiocyanate (FITC)-conjugated annexin antibody (5 µl) and prodium iodide (PI) solution (5 µl) were added into prepared cell suspension, and the mixtures were incubated for 30 minutes at room temperature in dark place. The cell suspension was analyzed using the flow cytometry.

### Tumourigenicity Assay in Nude Mice

2×10^6^ tumor cells were injected subcutaneously into the flanks of 6-week-old male BALB/c nude mice. Animal experiment procedures were approved by the institutional animal ethic committee of Nanjing Medical University. Tumor dimensions were monitored twice a week by means of Vernier calipers and- tumor volume was calculated using the formula: tumor volume = π/6×(major axis)×(minor axis)^2^. The tumor progression kinetics was estimated by tumor size and volume. Sorafenib was prepared fresh daily just before gavage, by dissolving in cremorphor EL/95% ethanol/water (12.5∶12.5∶75) as described previously [19], [20]. The drug or vehicle control was administered by daily gavage at stepwise dose levels of 15, 30, and 60 mg/kg body weight for 4 weeks.

### Immunohistochemistry Assay

Paraffin-embedden xenograft tumor samples were cut into 4 µm thin slices. After deparaffinization and dehydration, endogenous peroxidase was inactivated by 2.5% H_2_O_2_ diluted in methanol. Tissue sections were subject to heat-induced antigen retrieval in 0.01 mmol/L sodium citrate buffer for 30 min. Slides were blocked in 20% (v/v) normal horse serum in phosphate buffer solution (PBS) and subsequently incubated with Ki-67 antibody (Santa Cruz). The immuno-signals were exhibited by a 3,3′-diaminoberzidine (DAB) substrate kit (Dako). All sections were counterstained with haematoxylin to show nuclei.

### Statistical Analysis

Data are presented as mean ± standard deviation (SD) from at least 3 independent experiments and analyzed using two-tailed Student’s *t* test. A ***P*** value of less than 0.05 was considered as statistically significant.

## Results

### Expression Profiles of Mitotic Spindle Checkpoint Genes in Human Hepatocellular Carcinoma

**Figure 1:**
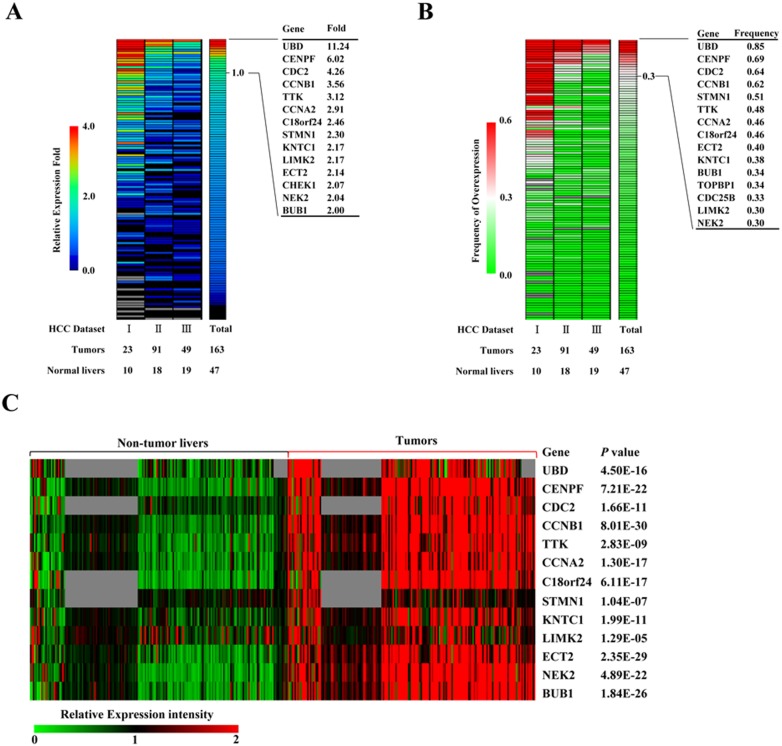
Expression profiles of selected mitotic spindle checkpoint genes in HCC microarray datasets. Heat maps of relative fold change (A) and frequency of overexpression (B) of gene expression (rows) in three HCC microarray datasets (columns). Number of normal liver and HCC tumor samples per data set are shown below. (C) As the heat map shown, 13 mitotic spindle checkpoint genes differentially expressed between HCC and adjacent non-tumorous livers in 4 independent HCC microarray datasets. Missing data are denoted in gray. Transcriptional signal intensity is normalized in these datasets analyzed. The significance of differential expression between HCC and adjacent non-tumorous liver tissue was evaluated using a two-tailed *t* test, and *P* values are provided.

First, we selected 137 known mitotic spindle checkpoint genes (**Table S1**) to examine their expression in 163 human HCC tumors relative to 47 normal livers in 3 expression datasets, GSE45114, GSE1898 and GSE4024. Entrez GeneIDs of the 137 genes were used to extract expression data from these microarray datasets. To determine the overexpressed genes in human HCC, the change fold and overexpression frequency of gene expression were calculated to identify markedly up-regulated genes in HCC tumors, compared with normal lives [21]. We defined the overexpression with no less than 2-fold level relative to normal livers and overexpression with no less than 30% of the patient population as the markedly overexpressed genes in HCCs (**Figure 1A and B**). As a result, 13 mitotic spindle checkpoint genes were considered as remarkable overexpression in human HCC. The 13 genes include cell cycle genes, such as CDC2, CCNB1, CCNA1, and kinase genes, such as TTK, LIMK2, NEK2, BUB1, and other genes UBD, CENPF, C18orf24, STMN1, KNTC1, ECT2 (**Table 1**).

**Table 1:** The 13 markedly overexpressed mitotic spindle checkpoint genes in HCC samples.

Gene	Description	ChromosomeLocation	Mutated/Total Samples*	Expression as acquired sorafenib-resistance†			
				Huh7R/Huh7		HepG2R/HepG2	
				fold ± s.d.	*P*-value	fold ± s.d.	*P*-value
UBD	ubiquitin D	6p21.3	0/312	1.25±0.38	0.615	0.99±0.31	0.708
CENPF	centromere protein F, 350/400 kDa	1q41	6/312	1.10±0.17	0.693	0.96±0.15	0.200
CDC2	cyclin-dependent kinase 1	10q21.1	0/312	1.01±0.20	0.278	7.12±2.05	0.007
CCNB1	cyclin B1	5q12	1/312	0.57±0.07	3.144E-04	0.72±0.15	0.009
TTK	TTK protein kinase	6q13-q21	0/312	4.40±0.77	0.002	4.09±0.98	0.006
CCNA2	cyclin A2	4q27	2/312	1.96±0.35	0.021	0.57±0.11	0.001
C18orf24	spindle and kinetochore associated complex subunit 1	18q21.1	0/312	1.00±0.21	0.545	0.99±0.18	0.099
STMN1	stathmin 1	1p36.11	1/312	1.15±0.25	0.985	0.93±0.14	0.051
KNTC1	kinetochore associated 1	12q24.31	6/312	6.04±0.68	2.252E-04	1.00±0.18	0.576
LIMK2	LIM domain kinase 2	22q12.2	0/312	1.07±0.11	0.427	0.60±0.08	4.139E-04
ECT2	epithelial cell transforming sequence 2 oncogene	3q26.1-q26.2	3/313	1.26±0.09	0.268	0.88±0.07	0.017
NEK2	NIMA-related kinase 2	1q32.2-q41	1/312	0.36±0.07	9.338E-05	1.47±0.18	0.040
BUB1	BUB1 mitotic checkpoint serine/threonine kinase	2q14	2/312	2.45±0.41	0.006	1.25±0.30	0.299

*shown is mutated number of total HCC samples stored in COSMIC (Catalogue of Somatic Mutations In Cancer, http://cancer.sanger.ac.uk/cancergenome/projects/cosmic/), which collects somatic mutation information in human cancers.

† Gene expression difference between these HCC cells and their derivative sorafenib-resistant cell sublines was evaluated by realtime quantitative PCR, where β-actin gene was used as an endogenous control. Statistical significance of gene expression difference was calculated using Student’s *t* test.

Next, the expression patterns of the 13 genes were further analyzed in a large number of HCC patients (184 cases) and their adjacent non-tumor tissues in 4 independent microarray datasets, GSE45114, GSE17856, GSE29721, and GSE22058. As expected, these genes were also significantly up-regulated in HCC patients compared with their adjacent non-tumorous livers (**Figure 1C** **and Figure S1**). In addition, we evaluated the somatic mutations of the 13 genes in HCC specimens by COSMIC (Catalogue of Somatic Mutations In Cancer) database. Surprisingly, somatic mutations of the 13 genes infrequently occurred, and only found in 0 to 6 cases in all 312 HCC patients analyzed (**Table 1**), indicating that they are primarily altered at the transcriptional levels in human HCCs.

### Role of the 13 Mitotic Checkpoint Genes in HCC Cells

Targeting mitotic spindle checkpoint genes was considered a promising therapeutic strategy in many human cancers [22], [23]. To investigate the role of these up-regulated mitotic checkpoint genes in HCC, we employed the RNA interference (RNAi) technique to evaluate the effect of the 13 genes on HCC cell proliferation. In order to ensure effective gene silence, 3 various siRNA duplexes targeting against each gene were pooled and delivered into HCC cell lines Huh7 and HepG2. We performed a quantitative RT-PCR to assess the RNAi knockdown efficiency mediated by these siRNAs against the 13 genes. The results showed that the mRNA levels of the 13 target genes were efficiently decreased to less than 60% by their relevant siRNAs in Huh7 and HepG2 cells (**Figure S2A**). Among the 13 marked up-regulated genes, 11 were with obvious inhibitory effects less than 75% cell viability relative to negative control siRNA (si-NC), as shown by cell viability assay in Huh7 and HepG2 (**Figure S2B**). To further confirm the inhibitory effects of the 11 genes on HCC cell proliferation, we performed soft agar colony formation assay by knocking them down by relevant siRNAs in Huh7 cells. Except for STMN1, KNTC1, these siRNAs against other 9 genes significantly inhibited anchor-independent colony formation of Huh7 cells (**Figure S2C**). These observations suggested that the 9 overexpressed mitotic checkpoint genes play an important role in HCC cell growth.

**Figure 2:**
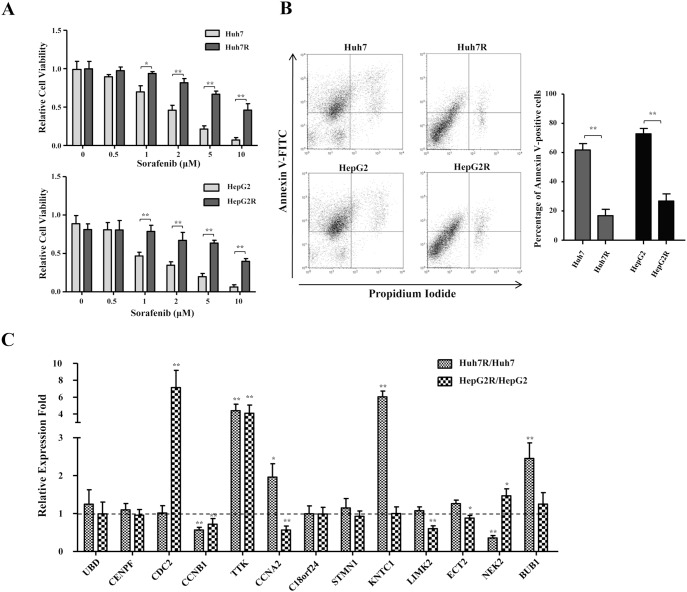
Expression analysis of the mitotic checkpoint genes in HCC cell lines and established sorafenib-resistant sublines. (A) The cytotoxic effects of sorafenib on sorafenib-naive and sorafenib-resistant cells of HCC cell lines Huh7 (upper panel) and HepG2 (lower panel). These cells were exposed to sorafenib at the indicated concentration for 24 h, and the cell viability was presented by the absorbance value at OD450 nm using CCK-8. *, ***P***<0.05, **, ***P***<0.01. (B) Scatter plots of fluorescence-activated cell sorting analysis with annexinV-FITC/PI staining in the sorafenib-naive and sorafenib-resistant cells of Huh7 and HepG2 exposed to 10 µM sorafenib (left panel). The histogram (right panel) shows the average percentages of annexin-positive cell population at three independent experiments ± standard deviation. (C) Expression analysis of the 13 mitotic spindle checkpoint genes in the sorafenib-naive and sorafenib-resistant cells of Huh7 and HepG2 using real-time PCR. Columns, mean (n = 3); bars, mean ± S.D. *, ***P***<0.05, **, ***P***<0.01.

As known, sorafenib is the only clinically approved drug, but HCC patients always develop acquired resistance to sorafenib. To identify appropriate therapeutic targets for sorafenib-resistant HCC, we established two sorafenib-resistant cell sublines derived from two HCC cell lines Huh7 and HepG2 by long-term exposure to sorafenib at stepwise increasing doses for a long time. As shown by cell viability assay (**Figure 2A**), Huh7R and HepG2R cells were significantly more viable than their progenitor cells in the presence of sorafenib in a dose-dependent way, indicating that these cells were more resistant to the cytotoxic effect of sorafenib. Subsequently, we analyzed apoptotic cell population using flow cytometry while these HCC cells were treated with 10 µM sorafenib. The sorafenib-resistant sublines Huh7R and HepG2R cells significantly exhibited less apoptotic cell populations than Huh7 and HepG2 cells, demonstrated by flow cytometry analysis (**Figure 2B**). These results indicated that the Huh7R and HepG2R cells acquired resistance to sorafenib. To investigate the correlation between the 13 overexpressed mitotic checkpoint genes and acquired sorafenib-resistance in HCC, we evaluated their expression changes between progenitors and derivative sorafenib-resistant sublines by quantitative PCR. The analysis of mRNA transcription level showed that the expression of these genes presented diversified patterns, but only TTK expression significantly increased in the both of two sorafenib-resistant sublines relative to their sorafenib-naive cells (**Figure 2C** **and** **Table 1**). The data suggested that TTK play a role in the acquisition of resistance to sorafenib in HCC.

### TTK was Frequently Up-regulated in HCC

**Figure 3:**
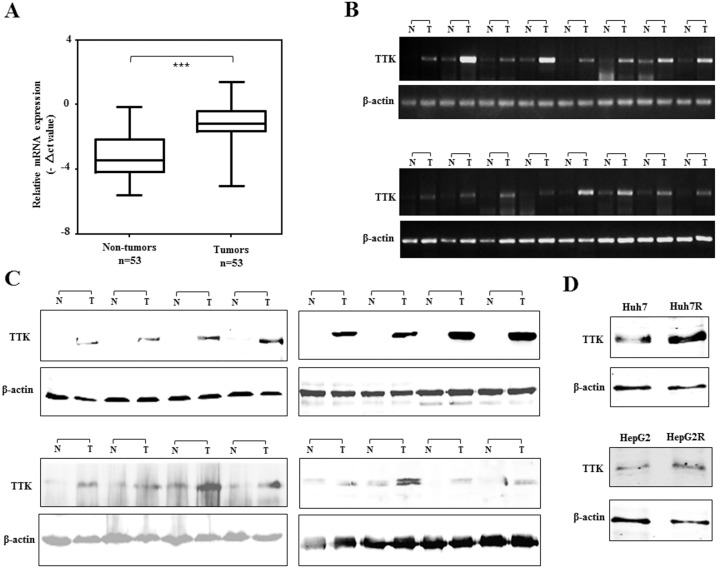
Expression profile of TTK in HCC specimens and cell lines. (A) Real-time RT-PCR analysis of TTK was performed on 53 paired HCCs and adjacent non-tumor livers. The relative mRNA level of TTK was normalized based on that of β-actin, and presented by box-whisker Plot. The line within each box represents the median −ΔCt value; the upper and lower edges of each box represent the 75th and 25th percentile, respectively; the upper and lower bars indicate the highest and lowest values, respectively. ***, ***P***<0.001. (B) Representative results of semi-quantitative RT-PCR of TTK from 16 pairs of HCC tumor (T) and corresponding non-tumor livers (N). (C) Western blot assay of 4 pairs of HCC specimens and adjacent non-tumor tissues with anti-TTK antibody, where β-actin was used as a loading control. (D) TTK protein expression was analyzed in Huh7, HepG2 cells and their sorafenib-resistant sublines by a western blotting assay.

To confirm the observation that TTK was obviously up-regulated in HCC samples of microarray datasets, the expression level of TTK was further evaluated in 53 paired human HCC specimens by real-time quantitative PCR. The resulting data showed that TTK mRNA expression was significantly increased in HCC as compared with adjacent non-cancerous livers (***P***<0.001, **Figure 3A**). Moreover, TTK was obviously up-regulated in 46 (86.8%) of 53 HCC specimens, whereas the PCR products of TTK transcript were rarely detected in adjacent non-cancerous livers, using a semi-quantitative PCR (**Figure 3B**). The up-regulation of TTK protein was confirmed in 16 paired HCC specimens with obvious up-regulated transcripts, as demonstrated by PCR assay (**Figure 3C**). However, the TTK upregulation was not statistically correlated with the gender, age, and tumor size (***P***>0.05, **Table S4**). To investigate the role of TTK upregulation in hepatocarcinogenesis, TTK expression signal was analyzed using the gene expression profiling of multi-stage samples from liver lesions, including 24 regenerative (cirrhotic) nodules (CN), 3 low-grade (LGDN), 12 high-grade dysplastic nodules (HGDN) and 10 early hepatocellular carcinomas (Early HCC) [24]. The data showed that TTK was significantly up-regulated in early HCC, compared with these CN, LGDN and HGDN liver lesion (**Figure S3**), suggested that TTK as a mitotic spindle checkpoint gene play an important role in early HCC. Moreover, TTK expression was observably increased when Huh7 and HepG2 acquired sorafenib-resistance, shown by western blotting assay (**Figure 3D**). This observation supports that TTK has a role in sorafenib-resistance of HCC cells.

### TTK Overexpression Promotes Cell Proliferation and Resistance to Sorafenib in HCC

**Figure 4:**
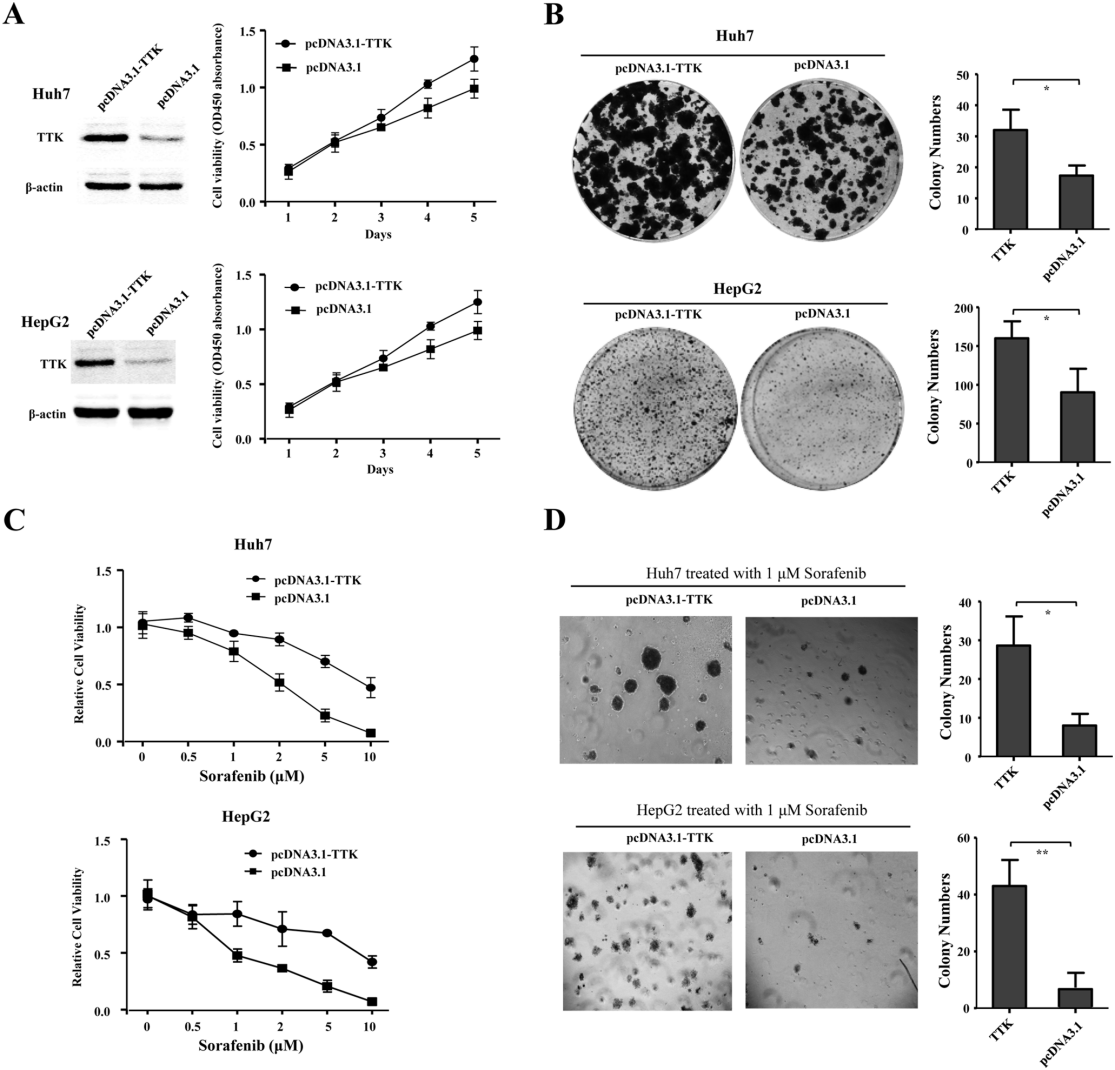
TTK overexpression promotes cell proliferation and resistance to sorafenib in HCC cells. (A) Cell growth curves were described according to cell viability for five days (right panel), when recombinant pcDNA3.1-TTK and empty vector were transiently transfected into Huh7 and HepG2 cells respectively, confirmed by western blotting assay, where β-actin was used as an internal reference (left panel). (B) Representative dishes of anchor-dependent colony formation of Huh7 and HepG2 cells were shown, where these cells were transfected with recombinant pcDNA3.1-TTK and empty vector (left panel). The numbers of colonies were counted and statistically analyzed using a two-tailed *t* test (right panel). (C) The cytotoxic effects of sorafenib at different concentrations (0.5, 1, 2, 5, 10 µM) on cell viability was shown, where pcDNA3.1-TTK and empty vector were transfected into Huh7 (upper panel) and HepG2 (lower panel) cells, respectively. (D) As shown were the microscopic fields of anchorage-independent colony formation assay of Huh7 and HepG2 cells treated with 1 µM sorafenib, upon TTK overexpression (left panel). The numbers of colonies were counted and statistically analyzed using a two-tailed *t* test (right panel). *, ***P***<0.05; **, ***P***<0.01.

To evaluate whether the up-regulation of TTK could contribute to hepatocarcinogenesis, Huh7 and HepG2 cells were transfected with recombinant construct pcDNA3.1-TTK and empty vector as control. The enforced TTK overexpression, as demonstrated by a western blot assay, significantly promoted cell growth (**Figure 4A**) and anchor-dependent colony formation (**Figure 4B**), as compared with that of those transfected with empty vector in Huh7 and HepG2 cells, respectively. We further investigated the cytotoxic effects of sorafenib on cell viability and anchor-independent colony formation when TTK was overexpressed in Huh7 and HepG2 cells. The resulting data showed that cytotoxic effect of sorafenib at different concentrations (0.5, 1, 2, 5, 10 µM) on cell viability was obviously restrained by the enforced TTK overexpression, as compared with that of those transfected with empty vector in Huh7 and HepG2 cells, respectively (**Figure 4C**). Similarly, TTK overexpression significantly resisted against 1 µM sorafenib treatment to promote anchor-independent colony formation, as compared with empty vector in Huh7 and HepG2 cells (**Figure 4D**). Collectively, the data suggested that the up-regulation of TTK could contribute to promote cell proliferation of HCC cells *in vitro*.

**Figure 5:**
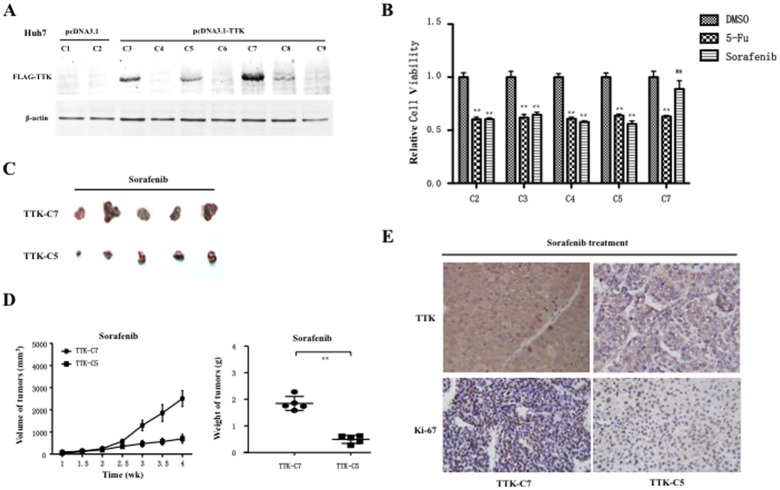
Tumorigenicity assay in nude mice of HCC cell subcolonies with TTK overexpression upon sorefanib administration. (A) Western blot analysis showed expression profile of exogenous FLAG-tagged TTK in those Huh7 cell subcolonies. (B) The relative cell viability or cytotoxicity assays of those offspring subcolonies of Huh7 cells with different TTK expression levels were performed, upon vehicle control DMSO, sorafenib or 5-Fluorouracil (5-FU) treatment, where the relative cell viability to DMSO control is shown in the histograms and statistically analyzed using a two-tailed *t*-test. *, ***P***<0.05; **, ***P***<0.01. (C) 2×10^6^ offspring cells of C7 subcolony were subcutaneously inoculated into the flanks of nude mice, whereas the same amount of cells of C5 subcolony were inoculated into the opposite flank of the same mice (n = 5). These xenograft tumors were removed from the mice and photographed. (D) Tumor size was estimated by serial calibration, where mean tumor volume (± sd) (left panel) and tumor weights were statistically analyzed using two-tailed *t*-test. **, ***P***<0.01 (right panel). (E) Representative pictures showed that immunohistochemistry staining assays of TTK and Ki-67 on xenograft tumours removed from the sorafenib-treated mice. All sections were counterstained with haematoxylin.

We established TTK-expressing Huh7 cells through stable transfection of FLAG-tagged TTK. Among 9 selected cell colonies, 3 (C3, C5, C7) exhibited discrepant TTK expression, other with few or no TTK expression compared with empty vector, as demonstrated by western blotting assay (**Figure 5A**). To investigate the role of TTK in sorafenib resistance, we further performed cell cytotoxicity assays when 5 subcolonies of Huh7 cells with different TTK expression levels, including 1 subcolony from cells transfected with empty vector and 4 subcolonies from cells transfected with TTK plasmid, were treated with 1 µM sorafenib or 4 mg/L 5-FU for 48 h, respectively. Expectedly, the relative cell viability was significantly inhibited by 5-FU in those subcolonies and without obvious differences among them. However, sorafenib treatment could lead to significant inhibition on cell viability of those subcolonies except for a subcolony with strong TTK expression (C7) (Figure 5B), strongly implying that TTK contributes to sorafenib resistance *in vitro*. To determine whether the sorafenib-resistant role of TTK depends on its proliferative effects. we subsequently investigated the sorafenib-resistant effects of enforced TTK expression on tumorigenicity *in vivo*. To reduce off promoting effects of TTK on proliferation, another subcolony (C5) from TTK-transfected cells was used as a mock control. Then, 2×10^6^ cells of the C7 subcolony were subcutaneously inoculated into the flanks of nude mice, whereas the same amount of C5 subcolony cells were inoculated into the opposite flank of the same mice (n = 4). The results showed that there were no significant differences of size and weight between two groups of xenograft tumors from C7 and C5 subcolony cells (**Figure S4**). Next, these cells of the 2 subcolony were inoculated into the flanks of nude mice (n = 5) in the same way, and these mice were administrated by sorafenib. Interestingly, we observed that these cells of C7 subcolony cells exhibited more enhanced tumorigenicity than those cells of C5 subcolony cells (**Figure 5C**). Moreover, the measurement data of size and weight of these xenograft tumors also showed that redundant TTK overexpression significantly promoted tumorigenesis upon sorafenib treatment (**Figure 5D**). To confirm molecularly tumorigenicity differences between two groups of subcolony cells with TTK-associated sorafenib-resistance, we evaluated TTK and Ki-67 a well-known proliferation marker) in these xenograft tumors with sorafenib treatment using immunohistochemical staining, where the intensity and proportion of cells with staining-positive signals in xenograft tumors were generally scored to the scale of 3+. Significantly, these tumors formed from C7 subcolony cells exhibited more TTK-positive signals than those formed from C5 subcolony cells; meanwhile, Ki-67-positive signals in tumors of C7 cells were enhanced as compared with those in tumors of C5 subcolony cells (**Figure 5E**). Overall, these results implied that TTK overexpression indeed contributes to sorafenib resistance. These findings demonstrate that TTK plays a dual roles in proliferation and sorafenib resistance during hepatocarcinogensis.

### TTK Knockdown Inhibits Cell Proliferation and Improves Anti-proliferative Effects of Sorafenib on HCC

**Figure 6:**
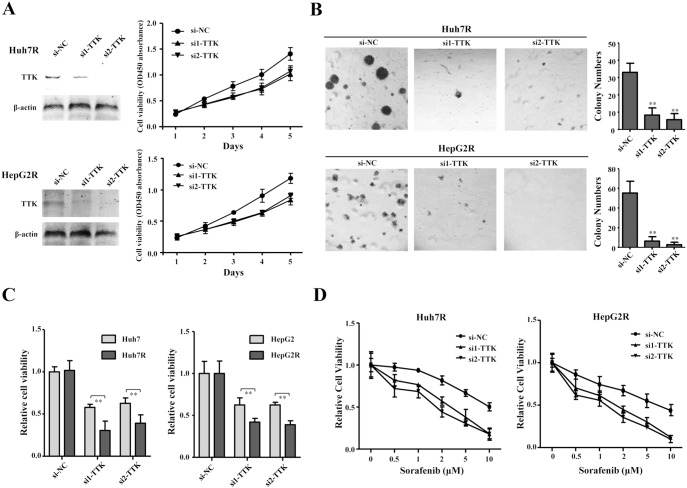
TTK knockdown suppresses cell proliferation and enhances cytotoxic effects of sorafenib on HCC cells. (A) TTK expression was obviously knocked down by the two siRNAs (si1-TTK, si2-TTK) in Huh7R and HepG2R (left panel), shown by western blotting assay and cell growth curves were shown according to cell viability for five days (right panel). (B) The microscopic fields shown were the anchorage-independent colony formation assay of Huh7R (upper panel) and HepG2R (lower panel) cells, where TTK was silenced by the two siRNAs (left panel). Colonies numbers were counted and statistically analyzed using a two-tailed *t* test. **, ***P***<0.01 (right panel). (C) Effects of TTK knockdown on cell viability of sorafenib-naive and sorafenib-resistant cells of Huh7 (left panel), HepG2 (right panel). Cell viability was presented by the absorbance value at OD450 nm using CCK-8 kit. Columns, mean (n = 3); bars, mean ± S.D. **, ***P***<0.01. (D) Cytotoxic effects of sorafenib at different concentrations (0.5, 1, 2, 5, 10 µM) on cell viability were shown, upon TTK knockdown in Huh7R (left panel) and HepG2R (right panel) cells, respectively.

To further confirm the roles of TTK in HCC, two efficient siRNAs (si1-TTK and si2-TTK) were used to silence endogenous TTK in sorafenib-resistant sublines of Huh7 and HepG2. The two siRNAs remarkably knocked the exogenous TTK down as compared with si-NC, demonstrated by western blotting assays in Huh7R and HepG2R cells (**Figure 6A**). We performed the cell growth curve and soft agar colony formation assay, upon TTK knockdown. The results showed that TTK knockdown inhibited cell growth curve of Huh7R and HepG2 cells (**Figure 6A**). Furthermore, TTK knockdown significantly suppressed anchor-independent colony formation of these sorafenib-resistant cells (**Figure 6B**). In addition, we employed the two siRNAs against TTK to confirm the more significant inhibition on cell viability of sorafenib-resistant cells than their progenitors (**Figure 6C**). Afterwards, we investigated the cytotoxic effects of sorafenib on cell viability when TTK was silenced in Huh7R and HepG2R cells. As expected, TTK knockdown improved the cytotoxic effects of sorafenib on cell viability of Huh7R and HepG2R cells, respectively (**Figure 6D**). These results suggest that TTK knockdown has the suppressive effects on proliferation and sorafenib resistance of these HCC cell models *in vitro*.

**Figure 7:**
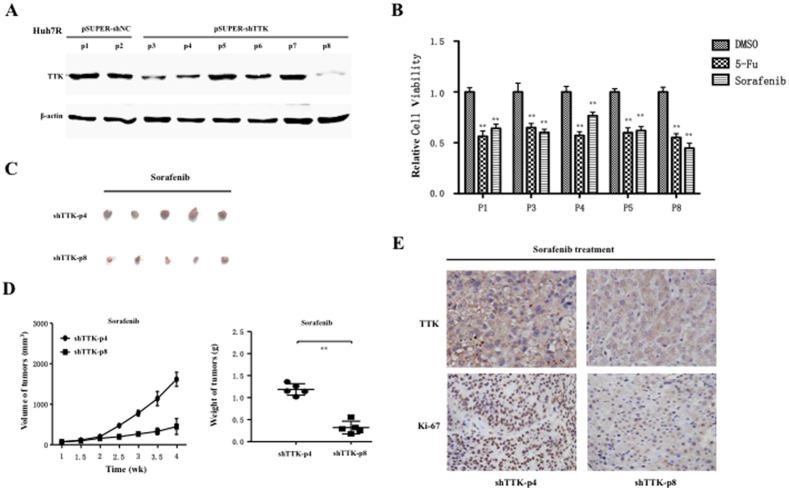
Tumorigenicity assay in nude mice of HCC subcolony cells with various TTK silence, upon sorefanib treatment. (A) Western blot analysis displayed expression profile of endogenous TTK in those Huh7R derivative subcolonies. (B) The relative cell viability or cytotoxicity assays of those Huh7R offspring cell subcolonies with varying TTK silence were performed, when these cells were treated with vehicle control DMSO, sorafenib or 5-Fluorouracil (5-FU) respectively. The relative cell viability to DMSO is shown in the histograms and statistically analyzed using a two-tailed *t*-test. **, ***P***<0.01. (C) 2×10^6^ cells of the two Huh7R offspring subcolonies (p8 and p4) were subcutaneously inoculated into the two flanks of nude mice, respectively (n = 5). These xenograft tumors were removed from the mice and photograph. (D) Tumor size was estimated by serial calibration, where mean tumor volume (± sd) (left panel) and tumor weights were statistically analyzed using two-tailed *t*-test. **, ***P***<0.01 (right panel). (E) Representative pictures showed that immunohistochemistry staining assays of TTK and Ki-67 on xenograft tumours removed from the sorafenib-treated mice. All sections were counterstained with haematoxylin.

To further investigate the role of TTK silence *in vivo*, we established offspring subclones of the Huh7R cell lines exhibiting stable knockdown of endogenous TTK by transfecting a pSUPER vector carrying the shRNA transformed by the sequence of si2-TTK above. Going through resistant selection, several offspring colonies (p3, p4, p8) with various TTK silence and control colonies (p1, p5) demonstrated by western blotting assay (**Figure 7A**), were used to investigate the role of TTK knockdown in sorafenib resistance. First, we performed cell cytotoxicity assays when those subcolonies of Huh7R cells with varying TTK silence degrees were treated with 1 µM sorafenib or 4 mg/L 5-FU for 48 h, respectively. As expected, the relative cell viability was significantly inhibited by 5-FU in those subcolonies and without obvious differences among them. Although sorafenib treatment also could lead to significant inhibition on the relative cell viability of those subcolonies, we observed the significant difference between p4 and p8 subcolonies with different TTK silence (Figure 7B). This finding implied that TTK knockdown could increase the sensitivity to sorafenib *in vitro*.

To validate this implication, next we investigated *in vivo* tumorigenicity of these subcolonies of Huh7R cells. These cells of p8 subcolony with remarkable TTK silence were subcutaneously inoculated into the flanks of nude mice, whereas those cells of p4 subcolony with moderate TTK silence were inoculated into the opposite flank of the same mice (n = 4). There were no significant differences of size and weight between two groups of xenograft tumors formed from p4 and p8 subcolony cells (**Figure S5**). Subsequently, these cells of the 2 subcolony were inoculated into the flanks of nude mice (n = 5), which were administrated by sorafenib. Intriguingly, the tumorigenicity of these cells of p8 subcolony reduced compared with those cells of p4 subcolony, upon sorafenib treatment (**Figure 7C**). Furthermore, the size and weight of these xenograft tumors also showed that TTK downregulation significantly suppressed tumorigenesis when sorafenib was administrated on mice with tumor burden (**Figure 7D**). Furthermore, we also examined TTK and Ki-67 in these sorafenib-treated xenograft tumors. The results showed these tumors formed from p8 subcolony cells had less staining signals of TTK than those formed from p4 subcolony cells, while Ki-67-positive signals in tumors of p8 cells were significantly reduced as compared with those in tumors of p4 subcolony cells (**Figure 7E**). These data demonstrated that TTK knockdown could contribute to promote sensitivity to sorafenib, and TTK had the dual roles in proliferation and sorafenib resistance. In all, our findings suggest that TTK is a potential therapeutic target, especially for human hepatocellular carcinoma.

## Discussion

As known, inhibition of the mitotic checkpoint is fatal to human cancer cells, and represents an attractive anti-cancer strategy. In this study, we used the publicly available HCC microarray datasets to analyze the expression of 137 selected mitotic spindle checkpoint genes in a large number of samples of liver cancers. Upon the defined overexpressed fold and frequency, we identified the 13 markedly up-regulated genes in human HCC specimens as candidates for further investigations. Among these candidates shown in **Table 1**, cell cycle regulator genes CDC2, CCNB1, CCNA1, are known to be dysregulated or activated in HCC and play an important role in human hepatocarcinigenesis [25]–[29]. UBD, also named FAT10, belongs to the ubiquitin-like protein family (Ubls). UBD, found to be overexpressed in 90% of human HCC, has a critical role in regulating diverse aspects of the pathogenesis of HCC [30]. CENPF, encoding a protein that associates with the centromere-kinetochore complex, exhibited coincidently amplification and overexpression in HCC. CENPF plays a role as common cancer-driver gene in human cancer [31]. C18orf24, whose formal symbol is SKA1 (spindle and kinetochore associated complex subunit 1), played an necessary role in the regulation of HCC cell proliferation and apoptosis [32]. All in all, these reports conform to our analysis results of microarray data.

We further investigated the role of these markedly up-regulated genes in cell proliferation and acquired sorafenib-resistance of HCC cell lines. Interestingly, many candidates including UBD, CENPF, CDC2, CCNB1, TTK, CCNA2, C18orf24, ECT2 and BUB1 were responsible for maintaining HCC cell proliferation, implying that these genes and protein products could be considered as potential therapeutic targets for HCC. In this study, we established two HCC cell sublines with sorafenib-resistance phenotype and evaluated the expression of these candidate genes in these sorafenib-naive and sorafenib-resistant HCC cells. We found that TTK, which encodes a dual specificity protein kinase essential for chromosome alignment and centrosome duplication [33], exhibited significantly increased expression in sorafenib-resistant HCC cells compared with their progenitor cells. TTK, also known as MPS1, redirected several key proteins to kinetochores to control mitotic spindle checkpoint [34], [35]. It has been found aberrantly overexpressed or mtated in a wide range of human tumors, including glioblastoma [36], breast cancer [37], [38], colorectal cancer [39]. Here, we found that TTK was up-regulated in 86.8% of HCC specimens. More significantly, TTK expression increased in the two sorafenib-resistant sublines relative to their parent cells and could be required for sustaining cell proliferation and acquired sorafenib-resistance. Further experiments *in vitro* and *in vivo* showed that TTK played the role in proliferation and sorafenib resistance at various stages during hepatocarcinogensis. Evidences in tumorigenicity of HCC cells with stable TTK overexpression and knockdown indicated that TTK contribute to sorafenib resistance at later stage of hepatocarcinogenesis, which may explain that sorafenib was beneficial to most HCC patients and acquired resistance could only developed with prolong treatment.

Molecular mechanisms involving in resistance to sorafenib revealed by investigators included PI3K/Akt signaling pathway, JAK-STAT pathways, hypoxia-inducible pathways, epithelial-mesenchymal transition [17], [40], a positive modifier GRP78 for sorafenib resistance acquisition in HCC [41], signaling pathways controlled by EGFR and HER-3 restrict sorafenib effects both in naive and sorafenib-resistant HCC cells [42], *etc*. However, the mechanisms involving in TTK-associated resistance to sorafenib remained unclear. Nevertheless, this finding that Mps1/AKT and B-Raf/ERK signaling form an auto-regulatory negative feedback loop in melanoma cells prompts possible associations between TTK-mediated sorafenib resistance and PI3K/Akt signaling pathway [43]; However, possible molecular mechanism involving in the role of TTK during hepatocarcinogenesis is worth further investigations.

## Supporting Information

Figure S1 **Expression analysis of candidate mitotic checkpoint genes in 4 microarray datasets.** The lines within each box represents the median normalized expression value; the upper and lower edges of each box represent the 75th and 25th percentile, respectively; the upper and lower bars indicate the highest and lowest values determined, respectively. Statistical analysis was performed by a two-tailed *t*-test.
(TIF)

Figure S2 **Roles of these siRNAs against the markedly overexpressed mitotic checkpoint genes in HCC cells.** (A) The efficiency of target gene knockdown by these siRNAs was assessed using real-time PCR in Huh7 (upper) and HepG2 (lower) cells. (B) The effect of these siRNAs against the markedly overexpressed mitotic checkpoint genes on cell viability of Huh7 (upper) and HepG2 (lower) cells. (C) As shown were representative presentations of anchor-independent colony formation assay of Huh7 cells cultured in medium containing soft agar (left), where the numbers of colonies containing 50 cells were counted and the significance were calculated by a two-tailed t test. *, ***P***<0.05. si-NC was used as a negative control, and these histograms showed the mean values of three independent experiments ± standard deviation.
(TIF)

Figure S3 Scatter plots of TTK expression analysis in liver samples, including 24 cirrhotic nodules (CN), 3 low-grade (LGDN), 12 high-grade dysplastic nodules (HGDN) and 10 early hepatocellular carcinomas (Early HCC). *, ***P*** <0.05; **, ***P*** <0.01; ***, ***P*** **<0.001.**
(TIF)

Figure S4 **Tumorigenicity assay of the 2 offspring subcolonies (C5, C7) of Huh7 cells with different TTK overexpression.** (A) 2×10^6^ cells of Huh7 offspring colony with strong TTK expression (C7) were subcutaneously inoculated into the flanks of nude mice, whereas the same amount of another subcolony cells (C5) with moderate TTK level were inoculated into the opposite flank of the same mice (n = 5). These xenograft tumors were removed from the mice and photographed. (B) Tumor size was estimated by serial calibration, where mean tumor volume (± sd) (left panel) and tumor weights were statistically analyzed using two-tailed t-test. ns, not significant.
(TIF)

Figure S5 **Tumorigenicity assay of the 2 offspring subcolonies (p4, p8) of Huh7R cells with various TTK knockdown.** (A) 2×10^6^ cells of Huh7R offspring p8 subcolony were subcutaneously inoculated into the flank of nude mice, whereas the same amount of p4 subcolony cells were inoculated into the opposite flank of the same mice (n = 5). These xenograft tumors were removed from the mice and photographed. (B) Tumor size was estimated by serial calibration, where mean tumor volume (± sd) (left panel) and tumor weights were statistically analyzed using two-tailed t-test. ns, not significant.
(TIF)

Table S1 **137 mitotic spindle checkpoint genes.**
(XLS)

Table S2 **HCC microarray datasets used in this study.**
(XLSX)

Table S3 **The sequence of siRNAs and primers for the 13 mitotic checkpoint genes.**
(XLSX)

Table S4 **The expression of TTK verus clinical features.**
(XLSX)

## References

[1] Cormier JN, Thomas KT, Chari RS, Pinson CW (2006) Management of hepatocellular carcinoma. J Gastrointest Surg 10: 761–780. 16713550 10.1016/j.gassur.2005.10.006

[2] Masuzaki R, Omata M (2008) Treatment of hepatocellular carcinoma. Indian J Gastroenterol 27: 113–122. 18787282

[3] Palmer DH, Hussain SA, Smith AJ, Hargreaves S, Ma YT, et al (2013) Sorafenib for advanced hepatocellular carcinoma (HCC): impact of rationing in the United Kingdom. Br J Cancer 109: 888–890. 23880824 10.1038/bjc.2013.410 PMC3749577

[4] Carr BI, D’Alessandro R, Refolo MG, Iacovazzi PA, Lippolis C, et al (2013) Effects of low concentrations of regorafenib and sorafenib on human HCC cell AFP, migration, invasion, and growth in vitro. J Cell Physiol 228: 1344–1350. 23169148 10.1002/jcp.24291 PMC3582757

[5] Forner A, Llovet JM, Bruix J (2012) Hepatocellular carcinoma. Lancet 379: 1245–1255. 22353262 10.1016/S0140-6736(11)61347-0 PMC13537732

[6] Cheng AL, Kang YK, Chen Z, Tsao CJ, Qin S, et al (2009) Efficacy and safety of sorafenib in patients in the Asia-Pacific region with advanced hepatocellular carcinoma: a phase III randomised, double-blind, placebo-controlled trial. Lancet Oncol 10: 25–34. 19095497 10.1016/S1470-2045(08)70285-7

[7] Llovet JM, Ricci S, Mazzaferro V, Hilgard P, Gane E, et al (2008) Sorafenib in advanced hepatocellular carcinoma. N Engl J Med 359: 378–390. 18650514 10.1056/NEJMoa0708857

[8] Musacchio A (2011) Spindle assembly checkpoint: the third decade. Philos Trans R Soc Lond B Biol Sci 366: 3595–3604. 22084386 10.1098/rstb.2011.0072 PMC3203455

[9] Musacchio A, Ciliberto A (2012) The spindle-assembly checkpoint and the beauty of self-destruction. Nat Struct Mol Biol 19: 1059–1061. 23132380 10.1038/nsmb.2429

[10] Li Y, Benezra R (1996) Identification of a human mitotic checkpoint gene: hsMAD2. Science 274: 246–248. 8824189 10.1126/science.274.5285.246

[11] Cahill DP, Lengauer C, Yu J, Riggins GJ, Willson JK, et al (1998) Mutations of mitotic checkpoint genes in human cancers. Nature 392: 300–303. 9521327 10.1038/32688

[12] Kops GJ, Foltz DR, Cleveland DW (2004) Lethality to human cancer cells through massive chromosome loss by inhibition of the mitotic checkpoint. Proc Natl Acad Sci U S A 101: 8699–8704. 15159543 10.1073/pnas.0401142101 PMC423258

[13] Kops GJ, Weaver BA, Cleveland DW (2005) On the road to cancer: aneuploidy and the mitotic checkpoint. Nat Rev Cancer 5: 773–785. 16195750 10.1038/nrc1714

[14] Saeki A, Tamura S, Ito N, Kiso S, Matsuda Y, et al (2002) Frequent impairment of the spindle assembly checkpoint in hepatocellular carcinoma. Cancer 94: 2047–2054. 11932908 10.1002/cncr.10448

[15] Nishida N, Kudo M (2013) Recent advancements in comprehensive genetic analyses for human hepatocellular carcinoma. Oncology 84 Suppl 193–97. 23428866 10.1159/000345897

[16] Nakagawa H, Shibata T (2012) Comprehensive genome sequencing of the liver cancer genome. Cancer Lett. 10.1016/j.canlet.2012.10.035 23142287

[17] Chen KF, Chen HL, Tai WT, Feng WC, Hsu CH, et al (2011) Activation of phosphatidylinositol 3-kinase/Akt signaling pathway mediates acquired resistance to sorafenib in hepatocellular carcinoma cells. J Pharmacol Exp Ther 337: 155–161. 21205925 10.1124/jpet.110.175786

[18] Uchibori K, Kasamatsu A, Sunaga M, Yokota S, Sakurada T, et al (2012) Establishment and characterization of two 5-fluorouracil-resistant hepatocellular carcinoma cell lines. Int J Oncol 40: 1005–1010. 22179686 10.3892/ijo.2011.1300 PMC3584526

[19] Xin H, Zhang C, Herrmann A, Du Y, Figlin R, et al (2009) Sunitinib inhibition of Stat3 induces renal cell carcinoma tumor cell apoptosis and reduces immunosuppressive cells. Cancer Res 69: 2506–2513. 19244102 10.1158/0008-5472.CAN-08-4323 PMC2664264

[20] Chang YS, Adnane J, Trail PA, Levy J, Henderson A, et al (2007) Sorafenib (BAY 43-9006) inhibits tumor growth and vascularization and induces tumor apoptosis and hypoxia in RCC xenograft models. Cancer Chemother Pharmacol 59: 561–574. 17160391 10.1007/s00280-006-0393-4

[21] Mir SE, De Witt Hamer PC, Krawczyk PM, Balaj L, Claes A, et al (2010) In silico analysis of kinase expression identifies WEE1 as a gatekeeper against mitotic catastrophe in glioblastoma. Cancer Cell 18: 244–257. 20832752 10.1016/j.ccr.2010.08.011 PMC3115571

[22] Janssen A, Kops GJ, Medema RH (2011) Targeting the mitotic checkpoint to kill tumor cells. Horm Cancer 2: 113–116. 21475725 10.1007/s12672-010-0059-x PMC3056011

[23] Manchado E, Guillamot M, Malumbres M (2012) Killing cells by targeting mitosis. Cell Death Differ 19: 369–377. 22223105 10.1038/cdd.2011.197 PMC3278741

[24] Kaposi-Novak P, Libbrecht L, Woo HG, Lee YH, Sears NC, et al (2009) Central role of c-Myc during malignant conversion in human hepatocarcinogenesis. Cancer Res 69: 2775–2782. 19276364 10.1158/0008-5472.CAN-08-3357 PMC2680077

[25] Yu Y, Jiang X, Schoch BS, Carroll RS, Black PM, et al (2007) Aberrant splicing of cyclin-dependent kinase-associated protein phosphatase KAP increases proliferation and migration in glioblastoma. Cancer Res 67: 130–138. 17210692 10.1158/0008-5472.CAN-06-2478

[26] Yeh CT, Lu SC, Chen TC, Peng CY, Liaw YF (2000) Aberrant transcripts of the cyclin-dependent kinase-associated protein phosphatase in hepatocellular carcinoma. Cancer Res 60: 4697–4700. 10987270

[27] Li KK, Ng IO, Fan ST, Albrecht JH, Yamashita K, et al (2002) Activation of cyclin-dependent kinases CDC2 and CDK2 in hepatocellular carcinoma. Liver 22: 259–268. 12100577 10.1046/j.0106-9543.2002.01629.x

[28] Ito Y, Takeda T, Sakon M, Monden M, Tsujimoto M, et al (2000) Expression and prognostic role of cyclin-dependent kinase 1 (cdc2) in hepatocellular carcinoma. Oncology 59: 68–74. 10895070 10.1159/000012140

[29] Wang X, Meng X, Sun X, Liu M, Gao S, et al (2009) Wnt/beta-catenin signaling pathway may regulate cell cycle and expression of cyclin A and cyclin E protein in hepatocellular carcinoma cells. Cell Cycle 8: 1567–1570. 19411833 10.4161/cc.8.10.8489

[30] Liu L, Dong Z, Liang J, Cao C, Sun J, et al.. (2013) As an independent prognostic factor, FAT10 promotes hepatitis B virus-related hepatocellular carcinoma progression via Akt/GSK3beta pathway. Oncogene. 10.1038/onc.2013.236 23812429

[31] Kim HE, Kim DG, Lee KJ, Son JG, Song MY, et al (2012) Frequent amplification of CENPF, GMNN and CDK13 genes in hepatocellular carcinomas. PLoS One 7: e43223. 22912832 10.1371/journal.pone.0043223 PMC3418236

[32] Qin X, Yuan B, Xu X, Huang H, Liu Y (2013) Effects of short interfering RNA-mediated gene silencing of SKA1 on proliferation of hepatocellular carcinoma cells. Scand J Gastroenterol. 10.3109/00365521.2013.828774 24010405

[33] Fisk HA, Mattison CP, Winey M (2004) A field guide to the Mps1 family of protein kinases. Cell Cycle 3: 439–442. 14963409

[34] Tighe A, Staples O, Taylor S (2008) Mps1 kinase activity restrains anaphase during an unperturbed mitosis and targets Mad2 to kinetochores. J Cell Biol 181: 893–901. 18541701 10.1083/jcb.200712028 PMC2426934

[35] Maciejowski J, George KA, Terret ME, Zhang C, Shokat KM, et al (2010) Mps1 directs the assembly of Cdc20 inhibitory complexes during interphase and mitosis to control M phase timing and spindle checkpoint signaling. J Cell Biol 190: 89–100. 20624902 10.1083/jcb.201001050 PMC2911671

[36] Tannous BA, Kerami M, Van der Stoop PM, Kwiatkowski N, Wang J, et al (2013) Effects of the selective MPS1 inhibitor MPS1-IN-3 on glioblastoma sensitivity to antimitotic drugs. J Natl Cancer Inst 105: 1322–1331. 23940287 10.1093/jnci/djt168 PMC3760778

[37] Maire V, Baldeyron C, Richardson M, Tesson B, Vincent-Salomon A, et al (2013) TTK/hMPS1 is an attractive therapeutic target for triple-negative breast cancer. PLoS One 8: e63712. 23700430 10.1371/journal.pone.0063712 PMC3658982

[38] Daniel J, Coulter J, Woo JH, Wilsbach K, Gabrielson E (2011) High levels of the Mps1 checkpoint protein are protective of aneuploidy in breast cancer cells. Proc Natl Acad Sci U S A 108: 5384–5389. 21402910 10.1073/pnas.1007645108 PMC3069188

[39] Niittymaki I, Gylfe A, Laine L, Laakso M, Lehtonen HJ, et al (2011) High frequency of TTK mutations in microsatellite-unstable colorectal cancer and evaluation of their effect on spindle assembly checkpoint. Carcinogenesis 32: 305–311. 21163887 10.1093/carcin/bgq272

[40] Zhai B, Sun XY (2013) Mechanisms of resistance to sorafenib and the corresponding strategies in hepatocellular carcinoma. World J Hepatol 5: 345–352. 23898367 10.4254/wjh.v5.i7.345 PMC3724962

[41] Chiou JF, Tai CJ, Huang MT, Wei PL, Wang YH, et al (2010) Glucose-regulated protein 78 is a novel contributor to acquisition of resistance to sorafenib in hepatocellular carcinoma. Ann Surg Oncol 17: 603–612. 19830497 10.1245/s10434-009-0718-8

[42] Blivet-Van Eggelpoel MJ, Chettouh H, Fartoux L, Aoudjehane L, Barbu V, et al (2012) Epidermal growth factor receptor and HER-3 restrict cell response to sorafenib in hepatocellular carcinoma cells. J Hepatol 57: 108–115. 22414764 10.1016/j.jhep.2012.02.019

[43] Zhang L, Shi R, He C, Cheng C, Song B, et al (2013) Oncogenic B-Raf (V600E) abrogates the AKT/B-Raf/Mps1 interaction in melanoma cells. Cancer Lett 337: 125–132. 23726842 10.1016/j.canlet.2013.05.029

