# Supplementary figures and images for: Expression and Function Analysis of Mitotic Checkpoint Genes Identifies TTK as a Potential Therapeutic Target for Human Hepatocellular Carcinoma
Source: PLoS One. 2014 Jun 6;9(6):e97739. doi: 10.1371/journal.pone.0097739 (PMC4048189; doi:10.1371/journal.pone.0097739)

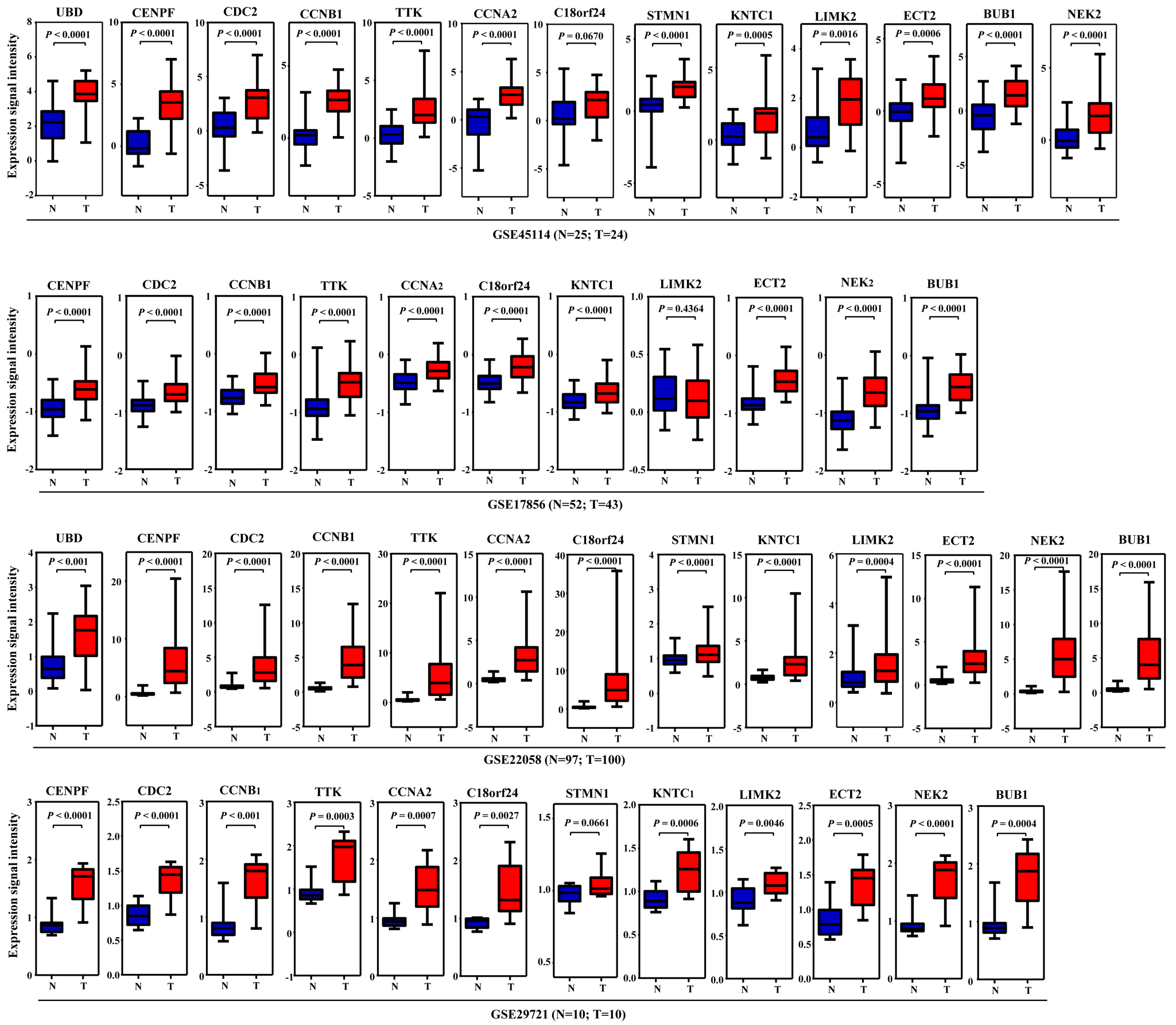

Supplement: Figure S1 — Expression analysis of candidate mitotic checkpoint genes in 4 microarray datasets. The lines within each box represents the median normalized expression value; the upper and lower edges of each box represent the 75th and 25th percentile, respectively; the upper and lower bars indicate the highest and lowest values determined, respectively. Statistical analysis was performed by a two-tailed t-test. (TIF) [file pone.0097739.s001.tif]

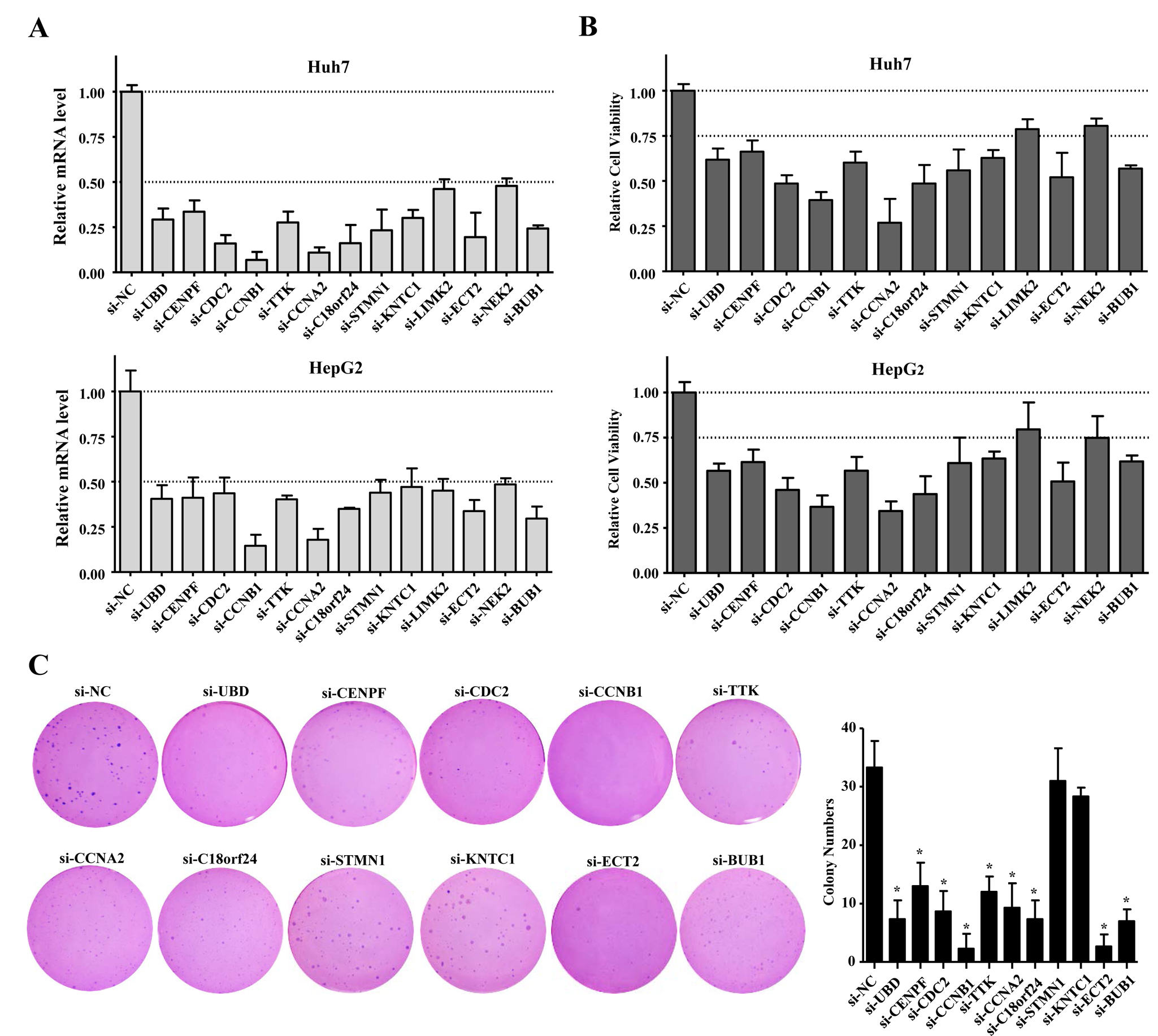

Supplement: Figure S2 — Roles of these siRNAs against the markedly overexpressed mitotic checkpoint genes in HCC cells. (A) The efficiency of target gene knockdown by these siRNAs was assessed using real-time PCR in Huh7 (upper) and HepG2 (lower) cells. (B) The effect of these siRNAs against the markedly overexpressed mitotic checkpoint genes on cell viability of Huh7 (upper) and HepG2 (lower) cells. (C) As shown were representative presentations of anchor-independent colony formation assay of Huh7 cells cultured in medium containing soft agar (left), where the numbers of colonies containing 50 cells were counted and the significance were calculated by a two-tailed t test. *, P<0.05. si-NC was used as a negative control, and these histograms showed the mean values of three independent experiments ± standard deviation. (TIF) [file pone.0097739.s002.tif]

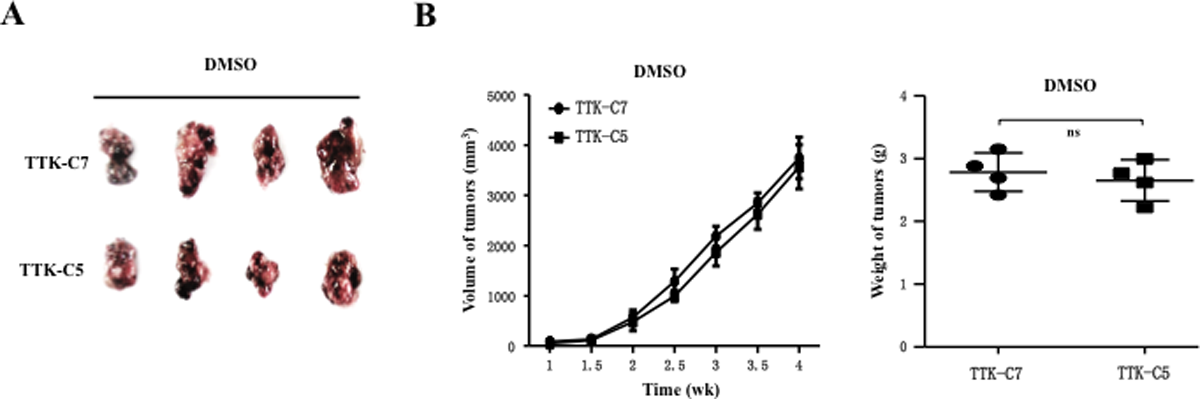

Supplement: Figure S4 — Tumorigenicity assay of the 2 offspring subcolonies (C5, C7) of Huh7 cells with different TTK overexpression. (A) 2×10^6^ cells of Huh7 offspring colony with strong TTK expression (C7) were subcutaneously inoculated into the flanks of nude mice, whereas the same amount of another subcolony cells (C5) with moderate TTK level were inoculated into the opposite flank of the same mice (n = 5). These xenograft tumors were removed from the mice and photographed. (B) Tumor size was estimated by serial calibration, where mean tumor volume (± sd) (left panel) and tumor weights were statistically analyzed using two-tailed t-test. ns, not significant. (TIF) [file pone.0097739.s004.tif]

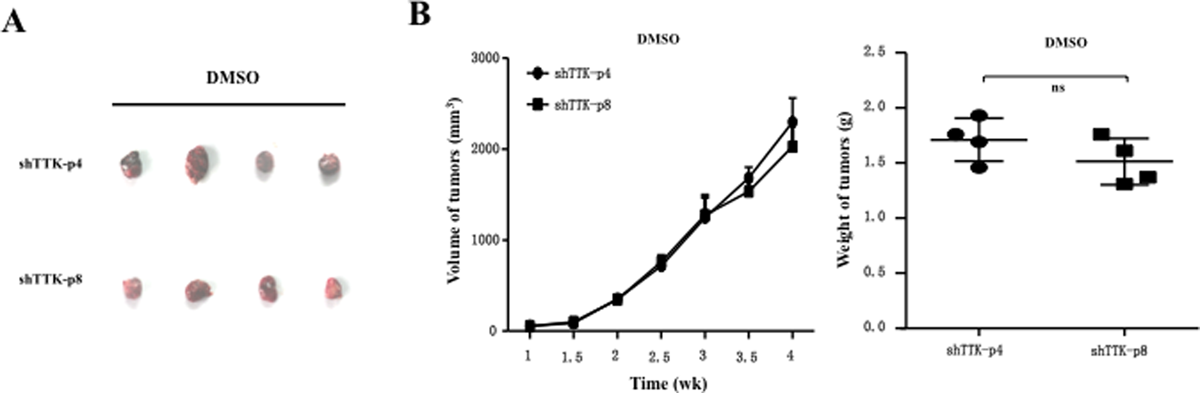

Supplement: Figure S5 — Tumorigenicity assay of the 2 offspring subcolonies (p4, p8) of Huh7R cells with various TTK knockdown. (A) 2×10^6^ cells of Huh7R offspring p8 subcolony were subcutaneously inoculated into the flank of nude mice, whereas the same amount of p4 subcolony cells were inoculated into the opposite flank of the same mice (n = 5). These xenograft tumors were removed from the mice and photographed. (B) Tumor size was estimated by serial calibration, where mean tumor volume (± sd) (left panel) and tumor weights were statistically analyzed using two-tailed t-test. ns, not significant. (TIF) [file pone.0097739.s005.tif]
